# Electrocardiographic changes caused by lithium intoxication in an elderly patient

**DOI:** 10.1186/s40064-015-1602-6

**Published:** 2016-01-04

**Authors:** Yiping Chen, Leilei Zheng, Weibo Liu, Huichun Li, Shaohua Yu, Qiaozhen Chen, Bin Pan, Hualiang Yu, Risheng Yu

**Affiliations:** Department of Psychiatry, Second Affiliated Hospital, Zhejiang University School of Medicine, 310009 Hangzhou, Zhejiang China; Department of Radiology, Second Affiliated Hospital, Zhejiang University School of Medicine, 310009 Hangzhou, Zhejiang China

**Keywords:** Lithium, Intoxication, Arrhythmia, Electrocardiography

## Abstract

Lithium intoxication can cause serious cardiac toxicity and is associated with electrocardiogram (ECG) changes. This paper described a case of a 76-year-old man who was lithium intoxicated and showed a variety of ECG abnormalities including sinus bradycardia, rapid atrial fibrillation, second-degree atrioventricular block and T wave changes. We monitored his ECGs during the after 3 days consecutively. After hemodialysis, his ECG abnormalities partially eased along with his serum lithium concentration decreased.

## Background

Generally, lithium is used as antimanic or mood-stabilizer, which has a narrow therapeutic range (0.8–1.2 mmol/L) (Griswold and Pessar 2000). Lithium overdose may induce various electrocardiographic (ECG) changes, such as junctional bradycaridia, complete heart block, first-degree atrioventricular block (Altinbas et al. 2014; Kayrak et al. 2010a; Singh et al. 2014). In addition, lithium-induced sinus node dysfunction has even been reported in its therapeutic range (Shetty et al. Shetty et al. 2013). Thus, individuals with underlying heart diseases are at higher risk even maintained with a dose of approximately 900–1200 mg/day (Griswold and Pessar 2000). We hereby reported a case of an elderly patient who was intoxicated severely after a long term of lithium treatment, his ECGs showed multiple changes including obvious sinus bradycardia, rapid atrial fibrillation, second-degree atrioventricular block, and T wave changes.

## Case description

The patient was a 76-year-old man who had a history of manic for 10 years, and had been treated with perphenazine in dose of 4–8 mg/day and lithium in dose of 500 mg/day for nearly 5 years. He had also suffered from hypertension for 30 years, which was well controlled by losartan 50 mg/day. One night a month ago, he became restless, irritable and full of self-praise. His hyperactivity and elevated mood were considered as the relapse of manic firstly. Then, we gave him lithium 1.0 g/day, perphenazine 14 mg/day and benzhexol 4 mg/day.

**Fig. 1:**
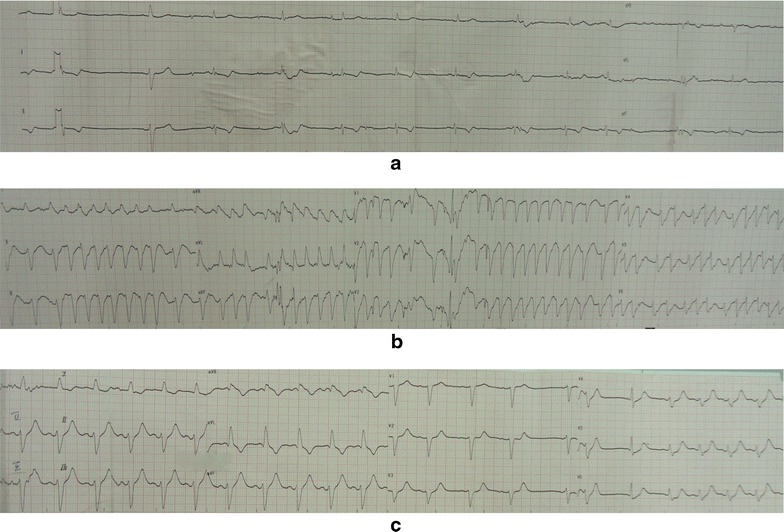
Electrocardiographic changes at lithium level of 5.0 mmol/L. **a** presented sinus bradycardia, sinus arrest, ventricular escape, ST-T segment changes, **b** presented rapid atrial fibrillation, intraventricular aberrant conduction, abnormal anteroseptal Q wave, **c** presented sinus arrhythmia, atrial escape, intraventricular block, ST-T segment changes

Four days before hospitalization, the patient presented anorexic and ataxic symptoms, but none of his dependents noticed that until he was unable to walk over the next 2 days. He was brought to emergency department for his limbs tremor and consciousness alert at night. On arrival, his vital signs were normal, but the laboratory test showed that his serum lithium concentration was 5.0 mmol/L, which indicated that he was severe lithium intoxication. Immediately, he was treated with hemodialysis. During the therapeutic process his heart rate dropped to 20 bpm, and ECG showed obvious sinus bradycardia, sinus arrest, ventricular escape, ST-T segment changes (Fig. 1a). After an administration of vasoactive drugs (dopamine 400 mg, aramine 100 mg, isoprenaline 2 mg), his heart rate was maintained between 45–55 bpm, and ECG showed atrial fibrillation(Fig. 1b), supraventricular tachycardia and spontaneous cardioversion.

**Fig. 2:**
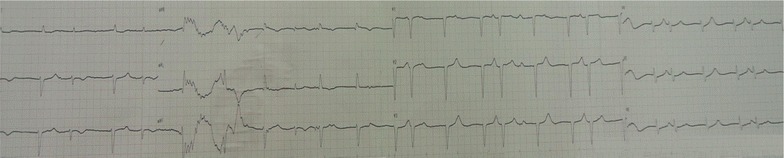
Electrocardiographic changes at lithium level of 1.3 mmol/L, second-degree atrioventricular heart block, junctional escape rhythm

**Fig. 3:**
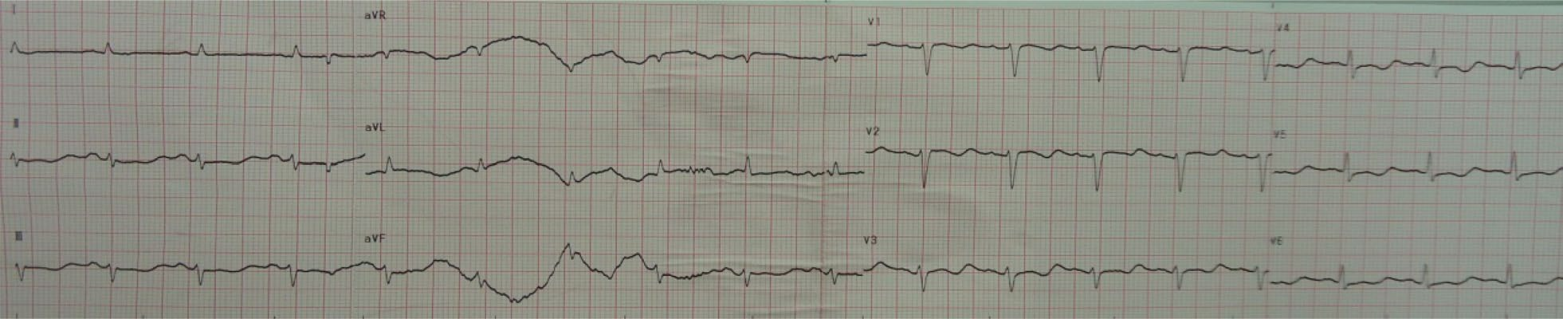
Electrocardiographic changes at lithium level of 1.1 mmol/L, sinus rhythm, T wave changes

After hemodialysis, the serum lithium concentration of this patient dropped to 1.3 mmol/L and the patient remained unresponsive to verbal stimulation, incoherent speech and high muscular tension. His ECG showed sinus arrhythmia (Fig. 1c), second-degree atrioventricular block, and junctional escape rhythm (Fig. 2). Hemodialysis had been maintained for 5 days. Over the next 3 days, his ECG showed that there were no obvious abnormalities except for T wave changes in the anterior leads (Fig. 3). The cardiac enzymes, serum potassium, serum calcium and renal clearance of this patient were within normal levels during his hospitalization. Seven days later, his symptoms were significant alleviated, his neurological examinations were all normal and orientation recovery.

## Discussion and evaluation

Lithium is now widely used for the treatment of schizoaffective disorder, depression, and bipolar disorder. Despite the clinical efficacy of lithium is known to all, however, we must use it with caution due to its narrow therapeutic index. Lithium intoxication can result in multisystem toxicity (Kansagra et al. 2014), just like Table 1 (Aruna 2009).

**Table 1:** Signs and symptoms of lithium intoxication in three degrees

Degree of intoxication	Signs and symptoms
Mild intoxication	Nausea/vomiting, diarrhea, drowsiness, lethargy, coarse hand tremor, and muscular weakness
Moderate intoxication	Nystagmus, ataxia, confusion, and myoclonus
Severe intoxication	Seizures, impaired consciousness, coma, and death

Beyond signs and symptoms above, the side effects of cardiac of lithium can be seen in a wide range of plasma concentrations (Mitchell and Mackenzie 1982; Waring 2007), there are kinds of ECG changes can be induced by lithium like Table 2 (Kayrak et al. 2010a; Mamiya et al. 2005; Mateer and Clark 1982; Mitchell and Mackenzie 1982; Oudit et al. 2007; Shetty et al. 2013; Singh et al. 2011).

**Table 2:** ECG changes induced by lithium

	ECG changes
Induced by lithium in a wide range of plasma concentrations	Dysfunction of sinus node
	Nonspecific T-wave flattening
	ST segment elevation
	Prolonged QT interval
	Ventricular tachycardia or ventricular fibrillation

Lithium is absorbed by gastrointestinal tract and is distributed in extracellular fluid initially. The elimination of lithium is exclusively by renal. Use of diuretic, renal impairment and hypertension patients with low salt diet all may impede the elimination of lithium, and lead to lithium accumulation in various tissues. The risk factors of lithium intoxication were diabetes insipidus, over 50 years old, hypothyroidism and deterioration in renal function (Behl et al. 2015; Shine et al. 2015). In this case, the patient had no hyponatremia and his renal clearance was normal. We speculated that his lithium intoxication was an acute-on-chronic ingestion of lithium. Because he was anorexic for several days, dehydration may result in unintentional lithium accumulation. Meanwhile, losartan is a kind of angiotensin-converting enzyme (ACE) inhibitors which can enhance lithium serum levels by increasing renal reabsorption in the proximal tubule (Haussmann et al. 2015). Specifically, ACE inhibitors can induce an acute natriuretic effect in rats or humans, which could lead to sodium depletion during chronic treatment with losartan. Meanwhile, a negative sodium balance is known to increase lithium reabsorption in renal (Aruna 2009). In addition, this patient also have perphenazine 14 mg/day before hospitalization for lithium together with little doses of antipsychotics can control the manic episode rapidly (Bowden 2004; Chou et al. 1999; Hollister 1972), as perphenazine is a kind of antipsychotics, and there is evidence that patients treated with lithium and antipsychotics had lower concentrating capacity than treated with lithium alone in renal, so it will increase the blood concentrations of lithium indirectly (Bucht and Wahlin 1980). Meanwhile, as Finley said in his report (Finley et al. 1995), patients treated with both lithium and an antipsychotic will have a neurotoxic reaction, and this neurotoxicity may be accounted for solely by the use of lithium (Ayd 1975). In a word, together with the negative influence of losartan and perphenazine, these two medicines may contribute to the lithium intoxication of this patient. Together with the matters above, Elderly and comorbid hypertension might also contributed to the development of lithium intoxication.

It had been proved that lithium is one of the most readily dialyzable toxins (Okusa and Crystal 1994), for its small volume of distribution, water solubility, and insignificant protein binding, which determined that hemodialysis can achieve far superior lithium clearance rates compared to other detoxification methods (Bayliss 2010). So, hemodialysis is highly effective in clearing lithium from the extracellular fluid and is recommended for the treatment of severe lithium intoxication (Goel et al. 2015). If the intoxication is founded on a chronic basis, hemodialysis may not gain such good outcome because lithium from intracellular fluid diffuses slowly to extracellular fluid. Whole-bowel was not given because the amounts of lithium remaining in the gastrointestinal track count for little in an acute-on-chronic intoxication case (Joshua et al. 2008).

In this case, the patient showed both ST-T segment changes and sinus arrest, he was ruled out for myocardial infarction and we found no abnormalities of serum potassium and calcium. We inferred several mechanisms might be concerned with lithium-related ECG changes. First, the competition of lithium with sodium, potassium, calcium, and magnesium ions could affect fluids and salts balance. Lithium’s interaction with sodium–calcium exchanger and Na/K pump could induce the physiology of cellular membrane changed. Lithium can decrease the intracellular potassium concentration, replace intracellular calcium and cause hypercalcemia (Albert et al. 2013), reduce the depolarization rate and electrical impulse propagation, and induce various ECG changes especially those related to the process of myocardial repolarization such as nonspecific ST segment and T wave changes. Second, lithium can also induce the sinus node dysfunction via its blockade of the voltage-gated sodium channels, which would decrease the sensitivity of the sinus node to sympathetic stimulation and therefore increase the incidence of impulse generation failure (sinus arrest) (Kayrak et al. 2010b; Talati et al. 2009), which explain the sinus arrest in this patient. Because lithium from intracellular fluid can diffuses slowly to extracellular fluid, hemodialysis for this patient had been maintained for 5 days to prevent ‘rebound’ of lithium concentrations (Canan et al. 2008), and his ECGs normalized along with the serum lithium level decreased. Thus, we considered that his ECG changes were associated with lithium overdose.

We didn’t give him lithium salts any more for the reason of he was 76-year-old, and had a history of lithium intoxication, we gave him depakine 500 mg/day, olanzapine 1.25 mg/day instead, 5 years later, he is in stable condition, and have no adverse reaction.

## Conclusions

In conclusion, we should bear in mind that lithium may interact with kinds of medicine, and result in various consequences; Various of ECG changes could be associated with lithium intoxication especially in elderly patients. It is important that the early measurement of serum lithium level both in emergency department and out-patient department is a good prevention for lithium intoxication.

## Abbreviations

ECG: electrocardiogram
ACE: angiotensin-converting enzyme
